# Advanced lung cancer inflammation index (ALI) predicts prognosis of patients with gastric cancer after surgical resection

**DOI:** 10.1186/s12885-022-09774-z

**Published:** 2022-06-21

**Authors:** Xin Zhang, Danfang Wang, Tuanhe Sun, Wenxing Li, Chengxue Dang

**Affiliations:** 1 Department of Oncology Surgery, First Affiliated Hospital of Xi’an Jiaotong University, 277 West Yanta Road, Xi’an, Shaanxi 710061 P. R. China; 2 Department of Xi’an Medical University, Xi’an, China

**Keywords:** Gastric cancer, ALI, Inflammation, Gender, Prognosis

## Abstract

**Introduction:**

Advanced lung cancer inflammation index (ALI) has been implicated in the prognosis of many types of tumors. But few studies elucidate its role in gastric cancer (GC).

**Materials and methods:**

We consecutively recruited 615 GC patients who underwent radical gastrectomy. Patients were grouped according to ALI status. Risk factors for overall survival (OS) and disease-free survival (DFS) in overall and sex-stratified cohorts were determined using multivariate cox regression analysis. We also compared survival differences between the two groups after one-to-one propensity score matching (PSM).

**Results:**

Patients with low ALI showed larger tumor size, more advanced TNM staging, shorter OS (median: 37 vs 42 months) and DFS (median: 37 vs 42 months) (all *P* < 0.001). Multivariate analysis showed that elevated ALI was independently associated with longer OS and DFS. After stratification by sex, low ALI was an independent risk factor for OS and DFS in male patients but not in female patients. But our further PSM analysis showed prognostic value of ALI in both male and female subgroups.

**Conclusion:**

Preoperative ALI is an independent prognostic factor for GC patients undergoing curative gastrectomy.

**Supplementary Information:**

The online version contains supplementary material available at 10.1186/s12885-022-09774-z.

## Introduction

Gastric cancer (GC) is the fifth most frequently diagnosed cancer and the third leading cause of cancer death worldwide [1]. Despite recent advances in surgery and chemotherapy, a large number of patients with GC recur after curative resection. There have been improvements in early detection, surgical treatment, chemotherapy, and molecularly targeted therapy in recent years, but the prognosis has been poor over the past decade [2].

Tumor-associated systemic inflammation plays a critical role in tumor cell development and metastasis [3]. Inflammation affects every step of tumorigenesis, from initiation, to promotion, to metastatic progression [4]. Previous studies have revealed that neutrophil-to-lymphocyte ratio (NLR) is closely related to tumor prognosis [5, 6]. Numerous reports describe the prognostic role of nutritional status and obesity in GC [7–9]. A novel inflammation-related marker, advanced lung cancer inflammation index (ALI), calculated from albumin, NLR and BMI (BMI × ALB/NLR), has been identified for the first time as a valid prognostic indicator in metastatic non-small cell lung cancer (NSCLC) [10]. ALI may have superior prognostic value as it reflects a combination of inflammation and nutrition status. Consistently, ALI as an independent predictor of many tumors, such as colon cancer [10, 11] and non-small cell lung cancer [12, 13], has been gradually revealed. However, there are few studies on the clinical significance of ALI in GC patient.

Given the above, we hypothesized that preoperative ALI could predict the prognosis of GC patients undergoing potentially curative resection. To test this hypothesis, we used a dataset of 615 GC cases and examined the association of preoperative ALI with clinicopathological factors and survival outcomes after radical resection in GC patients.

## Materials and methods

### Patients

615 primary GC patients who underwent radical gastrectomy between 2010 and 2017 were retrospectively enrolled. Patients were staged according to the tumor-node-metastasis (TNM) criteria (the eighth edition systems recommended by American Joint Committee on Cancer). Patient follow-up data were obtained through regular follow-up with a final follow-up time of June 2020. Overall survival (OS) was defined as the time interval between the date of radical surgery and the time of last follow-up or time of death, and disease-free survival (DFS) was defined as the time interval between the date of radical surgery and the time of last follow-up or time of recurrence. For OS, the endpoint event was death, and for DFS the endpoint event was tumor recurrence. The absence of any endpoint event up to the last follow-up was defined as censoring. Inclusion criteria: (1) All patients were initially diagnosed and had pathological evidence; (2) TNM stage I-III disease; (3) age ≥ 18 years; (4) R0 resection; (5) All clinical data are available. Exclusion criteria: (1) accompanying or secondary to other tumors; (2) Infection, inflammation, hematologic disease or taking medications that affect hematology 3 months before surgery; (3) history of radiotherapy or neoadjuvant chemotherapy; (4) Lost to follow-up. This study was approved by the Ethics Committee of First Affiliated Hospital of Xi’an Jiaotong University. Laboratory test was those nearest to the time of treatment. All methods were performed in accordance with the relevant guidelines and regulations.

### Evaluation of baseline characteristics

We collected gender, age at surgery, height, weight, preoperative laboratory test (including peripheral blood cells and albumin) and pathological parameters (tumor location, T stage, N stage, TNM stage, histology, lymph nodes retrieval and tumor size). BMI was defined as weight (kg)/height (m)/height (m). Neutrophil–lymphocyte ratio (NLR) was defined as absolute neutrophil count divided by absolute lymphocyte count. ALI was calculated as follows: ALI = BMI (kg/m^2^) × Albumin (g/dl)/NLR. Lymph node biopsy positive rate (LPR) was calculated by dividing the number of tumor cell positive lymph nodes by the number of resected lymph nodes. Tumor histology was divided into undifferentiated type (including undifferentiated or poorly differentiated adenocarcinoma, mucinous carcinoma and signet ring cell carcinoma) and differentiated type (including well or moderately differentiated adenocarcinoma). We divided patients into two groups based on cutoff value of ALI obtained from receiver operating characteristic (ROC) curve.

### Statistical analysis

Cases were grouped according to ALI level. Categorical variates were presented as frequencies and percentages and compared using the chi-square test or Fisher exact test. Continuous non-normal variates were presented as the median and interquartile range (IQR) and compared with log-rank tests, while continuous normally distributed variates were presented as mean ± standard deviation and compared using Student’s *t-tests*. The cut-off for BMI was set to 25 kg/m^2^, and for albumin and NLR was obtained based on ROC analysis. Differences in OS and DFS were assessed by the log-rank test and visualized using the Kaplan–Meier method. 5-year survival rate was obtained from survival analysis table. Independent prognostic factors for OS and DFS were determined by multivariate Cox proportional hazards regression analysis and assessed by Wald’s test. Variables with *P* < 0.05 in univariate analysis were included in multivariate analysis. Considering the difference in BMI between men and women, multivariate Cox regression analysis was also conducted in cohort after stratification by gender. To eliminate the effect of confounding covariates on survival analysis, PSM was performed using one-to-one nearest neighbor matching. The matching tolerance was set at 0.02, and the predictors involved in the PSM model were age, gender, tumor location, tumor differentiation, TNM stage, and postoperative chemotherapy.

Statistical analysis and plotting were performed with SPSS Statistics (version 22.0, IL, USA), 2-sided *p* < 0.05 were considered statistical significantly.

## Result

### Demographic and clinicopathological features of patient

We retrospectively enrolled 615 patients, including 146 (23.7%) female and 201 (32.7%) over 65 years old. To assess the association between preoperative ALI and clinicopathological characteristic, patients were divided into two group according the optimal cut-off (39.77) of ALI. The cutoff value was obtained at the maximum Youden index with a sensitivity of 64.2% and a specificity of 51.7% (Supplementary Fig. S1A). Low ALI was found significantly associated with advanced TNM stage, larger tumor size, shorter OS and shorter DFS (all *P* < 0.001). There was no significant difference in gender, age, adjuvant chemotherapy, tumor histology, tumor location and lymph node positive rate on biopsy (LPR) between these two groups (Table 1).

**Table 1:** Demographic and baseline characteristics of the two patient groups (*N* = 615)

Characteristics	All	Low ALI	High ALI	*P* value
	(*N* = 615)	(*N* = 253)	(*N* = 362)	
Gender				0.434
Female	146(23.7)	56(22.1)	90(24.9)	
Age ≥ 65 years	201(32.7)	93(36.8)	108(29.8)	0.072
ALI	44.71(29.98–60.65)	27.09(20.39–32.76)	57.19(48.64–73.58)	< 0.001
BMI, kg/m^2^	22.04(20–24.56)	21.01(19.03–23.08)	22.81(20.83–25.23)	< 0.001
Albumin, g/l	38.76 ± 4.51	37.06 ± 4.84	39.95 ± 3.84	< 0.001
NLR	1.95(1.45–2.72)	2.88(2.36–3.78)	1.56(1.23–1.88)	< 0.001
T stage				< 0.001
T1	148(24.1)	44(17.4)	104(28.7)	
T2	46(7.5)	11(4.3)	35(9.7)	
T3	60(9.8)	28(11.1)	32(8.8)	
T4	361(58.7)	170(67.2)	191(52.8)	
N stage				0.043
N0	280(45.5)	108(42.7)	172(47.5)	
N1	87(14.1)	28(11.1)	59(16.3)	
N2	111(18)	49(19.4)	62(17.1)	
N3	137(22.3)	68(26.9)	69(19.1)	
TNM stage				< 0.001
I	170(27.6)	48(19)	122(33.7)	
II	74(12)	28(11.1)	46(12.7)	
III	371(60.3)	177(70)	194(53.6)	
Chemotherapy^a^				0.152
yes	378(61.5)	164(64.8)	214(59.1)	
no	237(38.5)	89(35.2)	148(40.9)	
Histology				0.341
differentiated	193(31.4)	74(29.2)	119(32.9)	
undifferentiated	422(68.6)	179(70.8)	243(67.1)	
Tumor location				0.135
proximal stomach	161(26.2)	75(29.6)	86(23.8)	
distal stomach	357(58)	135(53.4)	222(61.3)	
total stomach	97(15.8)	43(17)	54(14.9)	
OS, month	40(27–64)	37(16.5–57.5)	42(32–65)	< 0.001
DFS, month	40(25–63)	37(15–56)	42(31–65)	< 0.001
LPR	0.05(0–0.29)	0.09(0–0.34)	0.04(0–0.27)	0.051
Tumor size, cm	4(2.5–5.5)	5(3–6.5)	3.5(2.1–5)	< 0.001

Data are presented as mean and standard deviation or median and interquartile range. *ALI* Advanced lung cancer inflammation index, *BMI* Body mass index, *NLR* Neutrophil–lymphocyte ratio, *TNM* Tumor-node-metastasis, *OS* Overall survival, *DFS* Disease-free survival, *LPR* Lymph node positive rate on biopsy. ^a^Postoperative adjuvant chemotherapy

### Low ALI was an independent risk factor for OS and DFS of GC patients

**Fig. 1:**
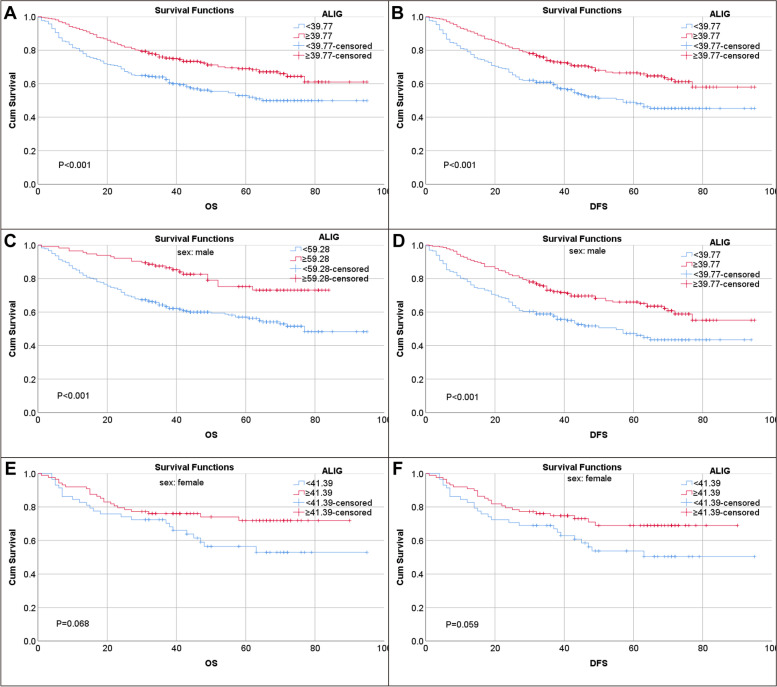
Kaplan–Meier survival curves of high ALI and low ALI groups for overall survival (OS) and disease-free survival (DFS). **A**, **B** survival curves for OS and DFS in the whole series. **C**, **D** survival curves for OS and DFS in male patients. **E**, **F** Survival curves for OS and DFS in female series

To explore the relationship between ALI and GC prognosis, Kaplan–Meier survival curve analysis was conducted according to ALI group. We found that patients with high ALI have significantly longer OS and DFS than low group (both *P* < 0.001) (Fig. 1A-B). Through the survival analysis table in terms of OS, we found that the 5-year survival rate of patients with high ALI was 63.5%, and that of patients with low ALI was 54.3%. As for DFS, 5-year survival rate was 61.1% and 50.4% for high ALI and low ALI, respectively. Subgroup analysis stratified by gender, we found that low ALI status was only significantly correlated with poor prognosis for OS (cutoff value: 59.28) and DFS (cutoff value: 39.77) in male GC patients (both *P* < 0.001) (Fig. 1C-D), whereas ALI status was not significantly associated with neither OS (cutoff value: 41.39, *P* = 0.068) nor DFS (cutoff value: 41.39, *P* = 0.059) in female patients (Fig. 1E-F). Notably, we performed ROC analysis based on endpoint events as well as subgroup populations to determine the optimal cutoff value (Supplementary Figs. S1 and S2). A series of cutoff values were shown in Supplementary Table 1.

To explore whether ALI is an independent prognostic factor, we performed univariate and multivariate COX regression analysis. Univariate analysis showed that elder age, low ALI, greater LPR, larger tumor size, proximal tumor location, advanced TNM stage and chemotherapy associated with shorter OS of GC patients. In multivariate analysis, elder age (HR: 1.936, 95%CI: 1.467–2.555, *P* < 0.001), high ALI (HR: 0.75, 95% CI: 0.567–0.992, *P* = 0.044), high LPR (HR: 5.619, 95% CI: 3.503–9.013, *P* < 0.001), larger tumor size (HR: 1.068, 95% CI: 1.015–1.124, *P* = 0.012), postoperative chemotherapy (HR: 0.455, 95% CI: 0.305–0.68, *P* < 0.001) and advanced TNM stage (TNM II stage: HR: 2.843, 95%CI: 1.323–6.107, *P* = 0.007, TNM III stage: HR: 7.626, 95%CI: 3.937–14.774, *P* < 0.001) were independent prognostic factors for OS (Table 2). In terms of DFS, elder age (HR: 1.912, 95%CI: 1.466–2.493, *P* < 0.001), high ALI (HR: 0.736, 95% CI: 0.564–0.961, *P* = 0.024), high LPR (HR: 5.431, 95% CI: 3.45–8.55, *P* < 0.001), larger tumor size (HR: 1.069, 95% CI: 1.017–1.123, *P* = 0.008), postoperative chemotherapy (HR: 0.479, 95% CI: 0.325–0.705, *P* < 0.001) and advanced TNM stage (TNM II stage: HR: 2.52, 95%CI: 1.251–5.076, *P* = 0.01, TNM III stage: HR: 6.358, 95%CI: 3.471–11.648, *P* < 0.001) were independently associated with DFS (Table 3). It is worth noting that the subgroup analysis by gender showed similar results to Kaplan–Meier survival curve analysis. In female patients, multivariate analysis showed no correlation between ALI status and OS or DFS (Supplementary Tables 2 and 4). In contrast, elevated ALI was significantly associated with longer OS in male patients (HR: 0.468, 95% CI: 0.291–0.751, *P* = 0.002) (Supplementary Table 3), so was DFS (HR: 0.664, 95% CI: 0.494–0.892, *P* = 0.007) (Supplementary Table 5).

**Table 2:** Univariate and multivariate analyses for overall survival of GC patients (*N* = 615)

Parameters	Univariate analysis			Multivariate analysis		
	HR	95%CI	*P* value	HR	95%CI	*P* value
Gender	1.188	0.86–1.64	0.296			
Age	1.723	1.317–2.255	< 0.001	1.936	1.467–2.555	< 0.001
ALI	0.571	0.438–0.744	< 0.001	0.75	0.567–0.992	0.044
LPR	11.208	7.541–16.658	< 0.001	5.619	3.503–9.013	< 0.001
Tumor size	1.154	1.114–1.196	< 0.001	1.068	1.015–1.124	0.012
Tumor location						
proximal stomach	1		< 0.001			0.444
distal stomach	0.684	0.504–0.929	0.015			0.255
full stomach	1.393	0.952–2.037	0.088			0.302
Histology	0.792	0.591–1.06	0.117			
TNM stage						
I	1			1		< 0.001
II	2.16	1.068–4.37	0.032	2.843	1.323–6.107	0.007
III	7.277	4.364–12.134	< 0.001	7.626	3.937–14.774	< 0.001
Chemotherapy	2.432	1.769–3.344	< 0.001	0.455	0.305–0.68	< 0.001

*HR* Hazard ratio, *CI* Confidence interval, *ALI* Advanced lung cancer inflammation index, *LPR* Lymph node positive rate on biopsy, *TNM* Tumor-node-metastasis. ALI was grouped according to cutoff value (39.77) obtained from ROC curve. The reference of gender, age, ALI, tumor location, histology and TNM stage was male, age < 65 years, low ALI, proximal stomach, undifferentiated and TNM stage I, respectively. LPR was analyzed as a continuous variable in univariate or multivariate analysis

**Table 3:** Univariate and multivariate analyses for disease-free survival of GC patients (*N* = 615)

Parameters	Univariate analysis			Multivariate analysis		
	HR	95%CI	*P* value	HR	95%CI	*P* value
Gender	1.207	0.886-1.645	0.232			
Age	1.693	1.308–2.19	< 0.001	1.912	1.466–2.493	< 0.001
ALI	0.562	0.436–0.724	< 0.001	0.736	0.564–0.961	0.024
LPR	10.666	7.281–15.625	< 0.001	5.431	3.45–8.55	< 0.001
Tumor size	1.152	1.113–1.193	< 0.001	1.069	1.017–1.123	0.008
Tumor location						
proximal stomach	1		< 0.001			0.424
distal stomach	0.68	0.508–0.91	0.009			0.215
full stomach	1.364	0.947–1.966	0.096			0.346
Histology	0.814	0.617-1.073	0.143			
TNM stage						
I	1		< 0.001	1		< 0.001
II	1.969	1.031–3.76	0.04	2.52	1.251–5.076	0.01
III	6.411	4.046–10.159	< 0.001	6.358	3.471–11.648	< 0.001
Chemotherapy	2.383	1.762–3.222	< 0.001	0.479	0.325–0.705	< 0.001

*HR* Hazard ratio, *CI* Confidence interval, *ALI* Advanced lung cancer inflammation index, *LPR* Lymph node positive rate on biopsy, *TNM* Tumor-node-metastasis. ALI was grouped according to cutoff value (39.77) obtained from ROC curve. The reference of gender, age, ALI, tumor location, histology and TNM stage was male, age < 65 years, low ALI, proximal stomach, undifferentiated and TNM stage I, respectively. LPR was analyzed as a continuous variable in univariate or multivariate analysis

Kaplan–Meier survival curve analysis was also performed for BMI, albumin and NLR (Supplementary Fig. S3A-I). Higher BMI, higher albumin and lower NLR is associated with gratified prognosis, which confirms the basis for the clinical significance of ALI. We also found that elevated BMI was only associated with improved survival in TNM stage III (*P* = 0.047), but not stage I and II (both *P* > 0.05) (Supplementary Fig. S3J-L). In view of the Kaplan–Meier survival analysis for DFS, we got similar results (Supplementary Fig. S4). To explore gender differences in ALI as a prognostic factor, after stratifying by gender, we performed a survival analysis on the above parameters. However, BMI, NLR and albumin were associated with OS and DFS in both male and female patients (all *P* < 0.05) (Supplementary Figs. S3 and S4).

### Patient characteristics and survival after propensity score matching

**Fig. 2:**
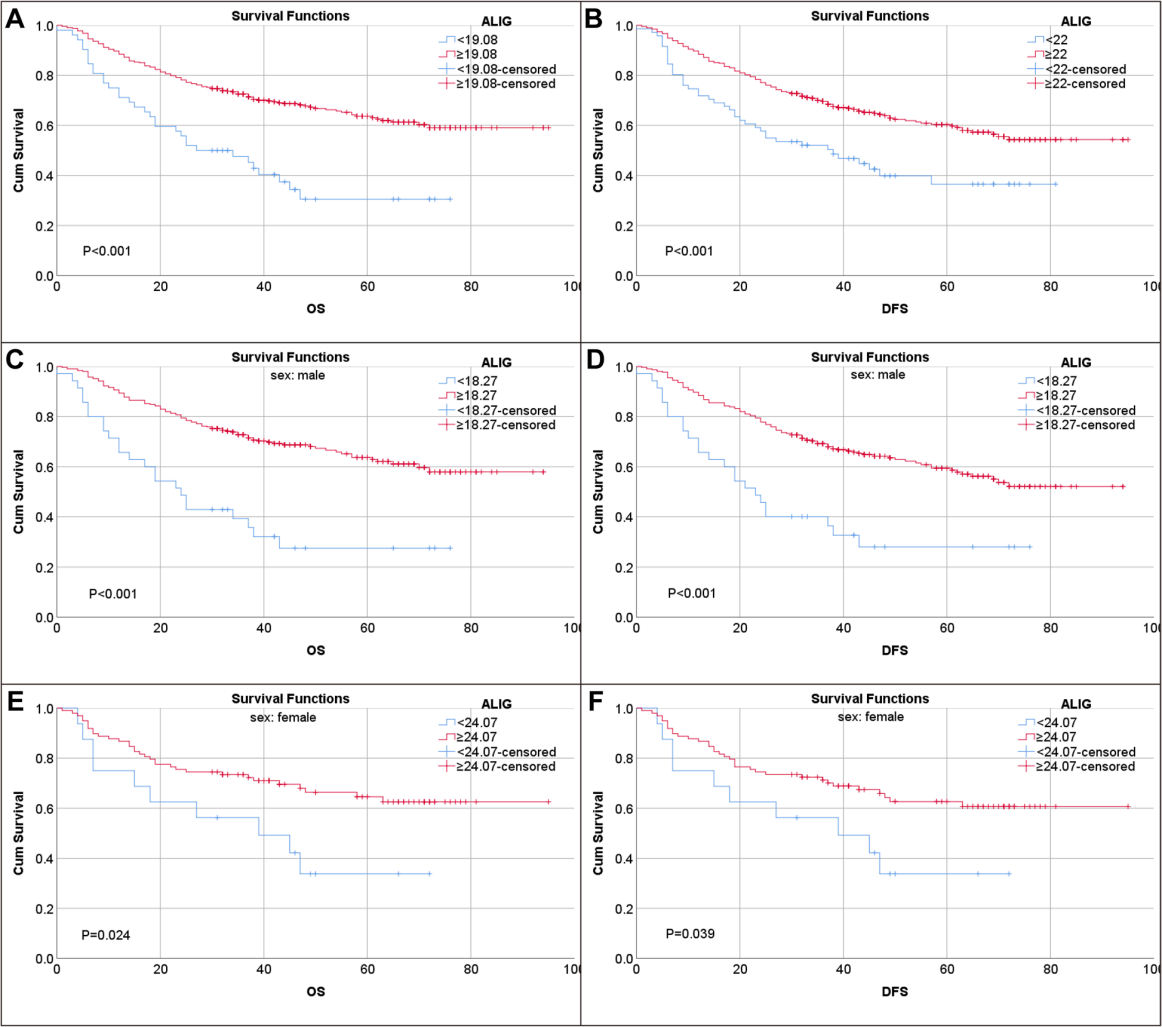
Survival curves for OS and DFS in the PSM cohort. **A**, **B** Survival curves dependents on ALI groups in all patients. **C**, **D** in male patients and **E**, **F** in female patients

To remove potential confounders for survival analysis, we performed PSM analysis, which adjusted for age, gender, tumor location, tumor differentiation, TNM stage, and postoperative chemotherapy. 460 patients were successfully matched. It can be seen that these adjusted factors mentioned above were not significantly different between the low and high ALI groups after PSM (Table 4). In this new dataset, we also obtained a series of cutoff values through ROC analysis (Supplementary Table 1, Supplementary Fig. S5). Kaplan–Meier survival curve analysis showed patients with high ALI still have significantly longer OS (*P* < 0.001) and DFS (*P* < 0.001) than control group (Fig. 2A-B). The survival analysis table showed that the 5-year overall survival rate was 30.5% for low ALI and 63.1% for high ALI. The 5-year DFS rate was 30.8% in low ALI and 59.4% in high ALI. Multivariate analysis showed independence of ALI for predicting OS (HR: 0.534, 95% CI: 0.362–0.785, *P* = 0.001) (Supplementary Table 6) and DFS (HR:0.599, 95% CI: 0.409–0.878, *P* = 0.009) (Supplementary Table 9). After subgroup analysis by gender, we found that ALI was significantly associated with OS and DFS regardless of gender (Fig. 2C-F) (Supplementary Tables 7, 8, 10 and 11).

**Table 4:** Demographic and baseline characteristics of the two patient groups (*N* = 460)

Characteristics	Overall	Low ALI	High ALI	*P* value
	(*N* = 460)	(*N* = 230)	(*N* = 230)	
Gender				0.388
Female	114(24.8)	53(23)	61(26.5)	
Age ≥ 65 years	161(35)	77(33.5)	84(36.5)	0.494
ALI	39.76(26.84–57.44)	26.88(19.78–32.35)	57.39(49.02–73.74)	< 0.001
BMI, kg/m^2^	21.9(19.59–24.34)	20.96(19–23.04)	22.71(20.76–25.07)	< 0.001
Albumin, g/l	38.51 ± 4.6	37.14 ± 4.83	39.89 ± 3.91	< 0.001
NLR	2.12(1.51–2.92)	2.91(2.38–3.83)	1.52(1.25–1.88)	< 0.001
TNM stage				0.797
I	102(22.2)	48(20.9)	54(23.5)	
II	53(11.5)	27(11.7)	26(11.3)	
III	305(66.3)	155(67.4)	150(65.2)	
Chemotherapy				0.548
yes	314(68.3)	160(69.6)	154(67)	
no	146(31.7)	70(30.4)	76(33)	
Histology				0.474
differentiated	135(29.3)	71(30.9)	64(27.8)	
undifferentiated	325(70.7)	159(69.1)	166(72.2)	
Tumor location				0.757
proximal stomach	121(26.3)	64(27.8)	57(24.8)	
distal stomach	260(56.5)	127(55.2)	133(57.8)	
total stomach	79(17.2)	39(17)	40(17.4)	
OS, month	39(25–61)	38(20.75–58.25)	41(30–64)	0.044
DFS, month	39(24–61)	38(19–58)	40(28.75–64)	0.027
LPR	0.08(0–0.31)	0.075(0–0.313)	0.09(0–0.31)	0.712
Tumor size, cm	4(2.8–6)	5(3–6.25)	3.5(2.5–5)	< 0.001

Propensity score-matched data adjusted for age, sex, tumor location, tumor differentiation, TNM stage, and postoperative chemotherapy. *ALI* Advanced lung cancer inflammation index, *BMI* Body mass index, *NLR* Neutrophil–lymphocyte ratio, *TNM* Tumor-node-metastasis, *OS* Overall survival, *DFS* Disease-free survival, *LPR* Lymph node positive rate on biopsy

## Discussion

In our current study, we investigated the prognostic value of ALI on long-term survival of patients with GC after radical resection. Our findings suggest that preoperative low ALI is an independent risk factor for OS and DFS. Preoperative ALI can provide convenient and inexpensive clinical decision-making guidance for patients undergoing radical gastrectomy.

Obesity has become a global health threat [14]. Worldwide, the cancer burden due to obesity is 11.9% in men and 13.1% in women [15]. Obesity also increases GC risk [16]. However, when the tumor has already occurred, the role of obesity on the tumor progression remains controversial. There is evidence that preoperative underweight and low nutritional index PNI were related to poor prognosis [17]. While preoperative overweight or mildly to moderately obese patients (BMI 23 to < 30 kg/m^2^) had better OS and disease-specific survival than normal-weight patients [18]. Paradoxically, studies have shown that postoperative BMI but not preoperative BMI is an independent prognostic factor for GC [19]. Not only that, breast cancer survivors with high BMI are at increased risk for developing second primary cancers [20]. The tumor-promoting effects of obesity are multifaceted, such as adipokines can promote tumor proliferation and survival [21], fatty acids produced from local adipose depots may feed nearby cancer cells [22]. Therefore, adipose tissue may promote tumorigenesis and progression and act as a risk factor. As tumors progress, cachexia is a major contributor to malnutrition, which is a determinant of tolerance to treatment and survival [23]. From this perspective, obesity can effectively offset the negative impact of malnutrition caused by cachexia, which may be the reason why obesity improves the prognosis of some tumors. In our current study, we found that high BMI is associated with favorable prognosis (Supplementary Figs. 3A and 4A). To explore whether the benefits and harms of obesity on tumors are related to tumor progression, we performed Kaplan–Meier analysis in subgroup based on TNM stage. Indeed, the clinical significance of BMI for outcome of GC was detected in TNM stage III, although no adverse effect of BMI on early stage tumors was found (Supplementary Figs. S3J-L and S4J-L). Since cancer patients are often accompanied by decreased nutritional status after surgery, we hypothesized that the benign prognostic effect of obesity may be due to nutritional factors.

There are several methods for assessing the nutritional status of cancer, of which serum albumin is one of the most commonly used. The association between malnutrition and cancer survival has received considerable attention in recent years. In fact, low serum albumin is associated with poor prognosis in gastrointestinal tumors [9, 24]. The mechanism of low serum albumin leading to poor tumor prognosis is complex. For example, low serum albumin level could impair the body’s natural defense mechanisms [25], reduce the efficiency of treatment options [26], as well as delayed recovery and increased mortality [27]. Various blood examination-based nutritional parameters have been implicated in tumor prognosis (prognostic nutritional index [28], albumin-globulin ratio [29] and c-reactive protein to albumin ratio [30]). In a word, albumin is closely related to tumor prognosis. Consistently, we verified that low albumin was significantly associated with shorter survival of GC patients (Supplementary Figs. S3D and S4D).

It is widely recognized that tumor-associated inflammation plays a crucial role in the development and progression of cancer [4]. Neutrophils, lymphocytes, monocytes, and platelets in peripheral blood routines are well-known inflammatory markers that may have prognostic roles in tumors. Inflammation-related markers derived therefrom, such as lymphocyte-monocyte ratio (LMR) [31], neutrophil–lymphocyte ratio (NLR) [5, 6], systemic immunity-inflammatory index (SII) [32, 33], platelet-lymphocyte ratio (PLR) [34] and systemic inflammation response index (SIRI) [35] on the survival outcome of GC patients have been reported. In line with this evidence, we found that patients with NLR ≥ 3.12 have significant shorter OS than those with low NLR (Supplementary Figs. S3G and S4G).

ALI is a recently described new marker of malignancy, which is specifically characterized by a comprehensive assessment of systemic inflammation and nutritional status. However, few studies have investigated ALI and survival after radical surgery in GC patients. Low ALI has been reported to be a negative predictor of long-term outcomes for overall and disease-free survival in GC patients [36]. Although preoperative ALI was not an independent prognostic factor for DFS in multivariate analysis. Considering the prognostic value of ALI for various tumors, especially lung cancer, and the evidence of BMI, albumin and NLR for tumor prognosis, we assumed ALI was a biomarker for disease status in GC. In our present study, we found that high ALI was significantly associated with longer OS (HR: 0.75, 95% CI: 0.567–0.992, *P* = 0.044) and DFS (HR: 0.736, 95% CI: 0.564–0.961, *P* = 0.024). Considering the difference in BMI between men and women, we conducted multivariate analysis in cohort stratified by gender. To our surprise, only LPR and tumor size remain significant for OS and DFS in female GC patients. While in male GC patients, together with younger age, lower LPR, early TNM stage and postoperative chemotherapy, elevated ALI was an independent protective factor for OS (HR: 0.468, 95% CI: 0.291–0.751, *P* = 0.002) and DFS (HR: 0.664, 95% CI: 0.494–0.892, *P* = 0.007). Gender differences in the prognostic role of ALI may be due to differences in BMI, albumin, and NLR on survival between men and women. On the other hand, it may also be caused by the fact of too few female cases (146 women) in our data set. To remove confounding variables from the survival analysis, we performed a PSM analysis. After adjusting for potential confounders, namely age, gender, tumor location, tumor differentiation, TNM stage, and postoperative chemotherapy, PSM analysis validated the prognostic effect of preoperative ALI in GC patients. As mentioned, we validated the favorable prognostic value of excess body weight, high albumin and low NLR. As ALI is a calculated indicator of inflammatory and nutritional status, a positive effect of high ALI on survival is expected. Indeed, we confirmed that ALI has significant effect on the prognosis of not only male but also female GC patients. 

Our study has some limitations. First of all, it is a single-center retrospective study. Secondly, ALI is a calculated indicator by BMI, albumin and NLR. Although NLR can reflect the level of systemic inflammation to a certain extent, its level is easily interfered by various factors, such as chronic inflammation, infection and drug effects. Moreover, as mentioned above, the prognostic value of various blood cell-derived inflammatory markers such as SIRI and PLR have been confirmed in recent years, and we did not compare the advantages of ALI with other inflammatory markers and nutritional markers. We also did not analyze the clinical significance of BMI, albumin and NLR in the prognosis of GC in detail.

In conclusion, we found that preoperative ALI was an independent factor for OS and DFS in GC patients undergoing radical surgery. Preoperative evaluation of ALI may help physicians determine postoperative oncological follow-up strategies and treatment strategies.

## Supplementary Information

**Additional file 1: Table S1.** Cutoff values for parameters in datasets before and after PSM. **Table S2.** Univariate and multivariate analyses for overall survival of female GC patients (N=146). **Table S3.** Univariate and multivariate analyses for overall survival of male GC patients (N=469). **Table S4.** Univariate and multivariate analyses for disease-free survival of female GC patients (N=146). **Table S5.** Univariate and multivariate analyses for disease-free survival of male GC patients (N=469). **Table S6.** Univariate and multivariate analyses for overall survival of GC patients after PSM (N=460). **Table S7.** Univariate and multivariate analyses for overall survival of male GC patients after PSM (N=346). **Table S8.** Univariate and multivariate analyses for overall survival of female GC patients after PSM (N=114). **Table S9.** Univariate and multivariate analyses for disease-free survival of GC patients after PSM (N=460). **Table S10.** Univariate and multivariate analyses for disease-free survival of male GC patients after PSM (N=346). **Table S11.** Univariate and multivariate analyses for disease-free survival of female GC patients after PSM (N=114). **Figure S1.** ROC analysis for the prediction of OS. AUC indicates the diagnostic power of ALI (A) in overall series, (B) in female subgroup and (C) in male subgroup. AUC indicates the diagnostic power of BMI (D) in overall series, (E) in female subgroup and (F) in male subgroup. AUC indicates the diagnostic power of ALB (G) in overall series, (H) in female subgroup and (I) in male subgroup. AUC indicates the diagnostic power of NLR (J) in overall series, (K) in female subgroup and (L) in male subgroup. Respective cutoff values obtained at the maximum Youden index was used to divide them into two groups. ROC, receiver operating characteristic curve; OS, overall survival; AUC, area under the ROC curve; ALI, advanced lung cancer inflammation index; BMI, body mass index; ALB, albumin; NLR, neutrophil-lymphocyte ratio. **Figure S2.** ROC analysis for the prediction of DFS. AUC indicates the diagnostic power of ALI (A) in overall series, (B) in female subgroup and (C) in male subgroup. AUC indicates the diagnostic power of BMI (D) in overall series, (E) in female subgroup and (F) in male subgroup. AUC indicates the diagnostic power of ALB (G) in overall series, (H) in female subgroup and (I) in male subgroup. AUC indicates the diagnostic power of NLR (J) in overall series, (K) in female subgroup and (L) in male subgroup. Respective cutoff values obtained at the maximum Youden index was used to divide them into two groups. DFS, disease-free survival. **Figure S3.** Kaplan-Meier survival analysis of BMI, ALB and NLR for the prediction of OS. Kaplan-Meier survival curves of BMI (A) in all patients, (B) in female patients and (C) in male patients. Kaplan-Meier survival curves of ALB (D) in all patients, (E) in female patients and (F) in male patients. Kaplan-Meier survival curves of NLR (G) in all patients, (H) in female patients and (I) in male patients. The respective cutoff values were obtained from the ROC curves of the subgroups. Both cutoff values and statistical significance are shown in figure. (J-L) Kaplan-Meier survival curves of BMI in groups stratified by TNM stage (P=0.205, 0.344 and 0.047, respectively). **Figure S4.** Kaplan-Meier survival analysis of BMI, ALB and NLR for the prediction of DFS. Kaplan-Meier survival curves of BMI (A) in all patients, (B) in female patients and (C) in male patients. Kaplan-Meier survival curves of ALB (D) in all patients, (E) in female patients and (F) in male patients. Kaplan-Meier survival curves of NLR (G) in all patients, (H) in female patients and (I) in male patients. The respective cutoff values were obtained from the ROC curves of the subgroups and shown in figure. (J-L) Kaplan-Meier survival curves of BMI in groups stratified by TNM stage (P=0.229, 0.211 and 0.047, respectively). **Figure S5.** ROC analysis for the prediction of OS and DFS in dataset after PSM. (A) ROC curve of ALI for the prediction of OS of all patients in this dataset; (B) ROC curve of ALI for the prediction of DFS of all patients in this dataset; (C) ROC curve of ALI for the prediction of OS of male patients; (D) ROC curve of ALI for the prediction of DFS of male patients; (E) ROC curve of ALI for the prediction of OS of female patients; (F) ROC curve of ALI for the prediction of DFS of female patients.

## Data Availability

The data generated in this study are available upon request from the corresponding author.
