# Supplementary material for: Advanced lung cancer inflammation index (ALI) predicts prognosis of patients with gastric cancer after surgical resection
Source: BMC Cancer. 2022 Jun 21;22:684. doi: 10.1186/s12885-022-09774-z (PMC9215041; doi:10.1186/s12885-022-09774-z)

**Advanced lung cancer inflammation index (ALI) predicts prognosis of male patients with gastric cancer after surgical resection**

Xin Zhang^1^, Danfang Wang^2^, Tuanhe Sun^1^, Wenxing Li^1^ and Chengxue Dang^1, #^

^1^Department of Oncology Surgery, First Affiliated Hospital of Xi’an Jiaotong University, Xi’an, China;

^2^Department of Xi'an Medical University, Xi’an, China;

^#^Corresponding Author: Prof. Chengxue Dang, Ph.D., M.D., Department of Surgical Oncology, First Affiliated Hospital Medical college Xi'an Jiaotong University, 277 West Yanta Road, Xi’an, Shaanxi 710061, P.R. China. Tel: 86-29-85324612, Fax: 86-29-85324612, E-mail: [dangchengxue@mail.xjtu.edu.cn](mailto:dangchengxue@mail.xjtu.edu.cn).

**Supplementary Table 1.** **Cutoff values for parameters in datasets before and after PSM**

| Parameters | | Death event^1^ | | | Recurrence event^2^ | | |
| --- | --- | --- | --- | --- | --- | --- | --- |
|  |  | overall | male | female | overall | male | female |
| Before PSM | ALI | 39.77 | 59.28 | 41.39 | 39.77 | 39.77 | 41.39 |
|  | BMI | 22.74 | 24.03 | 22.23 | 24 | 24.48 | 22.23 |
|  | ALB | 40.05 | 40.45 | 39.75 | 40.05 | 40.45 | 39.75 |
|  | NLR | 3.11 | 2.08 | 3.54 | 2.36 | 2.57 | 3.54 |
| After PSM | ALI | 19.08 | 18.27 | 24.07 | 22 | 18.27 | 24.07 |

^1^Cutoff values obtained by ROC analysis with death as the endpoint event; ^2^Cutoff values obtained by ROC analysis with tumor recurrence as the endpoint event. PSM, propensity score matching; ALI, advanced lung cancer inflammation index; BMI, body mass index; ALB, albumin; NLR, neutrophil-lymphocyte ratio.

**Supplementary Table 2. Univariate and multivariate analyses for overall survival of female GC patients (N=146)**

| Parameters | Univariate analysis | | | Multivariate analysis | | |
| --- | --- | --- | --- | --- | --- | --- |
|  | HR | 95%CI | P value | HR | 95%CI | P value |
| Age | 1.089 | 0.59-2.012 | 0.785 |  |  |  |
| ALI | 0.592 | 0.334-1.049 | 0.073 |  |  |  |
| LPR | 20.235 | 8.567-47.793 | <0.001 | 16.635 | 6.754-40.97 | <0.001 |
| Tumor size | 1.13 | 1.068-1.195 | <0.001 | 1.102 | 1.031-1.177 | 0.004 |
| Tumor location |  |  |  |  |  |  |
| proximal stomach | 1 |  | 0.08 |  |  |  |
| distal stomach | 1.018 | 0.445-2.325 | 0.967 |  |  |  |
| full stomach | 2.222 | 0.853-5.79 | 0.102 |  |  |  |
| Histology | 0.605 | 0.283-1.297 | 0.197 |  |  |  |
| TNM stage |  |  |  |  |  |  |
| I | 1 |  | 0.001 |  |  | 0.453 |
| II | 2.744 | 0.737-10.219 | 0.132 |  |  | 0.738 |
| III | 6.268 | 2.236-17.577 | <0.001 |  |  | 0.4 |
| Chemotherapy | 2.2 | 1.16-4.17 | 0.016 |  |  | 0.592 |

HR, hazard ratio; CI, confidence interval; ALI, advanced lung cancer inflammation index; LPR, lymph node positive rate on biopsy; TNM, tumor-node-metastasis. ALI was grouped according to cutoff value (41.39) obtained from ROC curve. The reference of gender, age, ALI, tumor location, histology and TNM stage was male, age<65 years, low ALI, proximal stomach, undifferentiated and TNM stage I, respectively. LPR was analyzed as a continuous variable in univariate or multivariate analysis.

**Supplementary Table 3. Univariate and multivariate analyses for overall survival of male GC patients (N=469)**

| Parameters | Univariate analysis | | | Multivariate analysis | | |
| --- | --- | --- | --- | --- | --- | --- |
|  | HR | 95%CI | P value | HR | 95%CI | P value |
| Age | 1.953 | 1.445-2.64 | <0.001 | 2.06 | 1.502-2.824 | <0.001 |
| ALI | 0.403 | 0.26-0.625 | <0.001 | 0.468 | 0.291-0.751 | 0.002 |
| LPR | 9.511 | 6.058-14.931 | <0.001 | 4.783 | 2.746-8.333 | <0.001 |
| Tumor size | 1.184 | 1.122-1.25 | <0.001 | 1.034 | 0.965-1.108 | 0.341 |
| Tumor location |  |  |  |  |  |  |
| proximal stomach | 1 |  | 0.002 |  |  | 0.532 |
| distal stomach | 0.65 | 0.463-0.91 | 0.012 |  |  | 0.733 |
| full stomach | 1.288 | 0.844-1.966 | 0.241 |  |  | 0.416 |
| Histology | 0.816 | 0.593-1.124 | 0.213 |  |  |  |
| TNM stage |  |  |  |  |  |  |
| I | 1 |  | <0.001 | 1 |  | <0.001 |
| II | 1.949 | 0.842-4.511 | 0.119 | 2.781 | 1.093-7.072 | 0.032 |
| III | 7.608 | 4.22-13.717 | <0.001 | 9.123 | 4.182-19.902 | <0.001 |
| Chemotherapy | 2.503 | 1.732-3.618 | <0.001 | 0.442 | 0.277-0.705 | 0.001 |

HR, hazard ratio; CI, confidence interval; ALI, advanced lung cancer inflammation index; LPR, lymph node positive rate on biopsy; TNM, tumor-node-metastasis. ALI was grouped according to cutoff value (59.28) obtained from ROC curve. The reference of gender, age, ALI, tumor location, histology and TNM stage was male, age<65 years, low ALI, proximal stomach, undifferentiated and TNM stage I, respectively. LPR was analyzed as a continuous variable in univariate or multivariate analysis.

**Supplementary Table 4. Univariate and multivariate analyses for disease-free survival of female GC patients (N=146)**

| Parameters | Univariate analysis | | | Multivariate analysis | | |
| --- | --- | --- | --- | --- | --- | --- |
|  | HR | 95%CI | P value | HR | 95%CI | P value |
| Age | 1.155 | 0.645-2.067 | 0.628 |  |  |  |
| ALI | 0.595 | 0.343-1.03 | 0.064 |  |  |  |
| LPR | 21.208 | 9.048-48.71 | <0.001 | 17.6049 | 7.204-43.015 | <0.001 |
| Tumor size | 1.123 | 1.062-1.186 | <0.001 | 1.093 | 1.024-1.167 | 0.007 |
| Tumor location |  |  |  |  |  |  |
| proximal stomach | 1 |  | 0.017 |  |  |  |
| distal stomach | 0.923 | 0.423-2.015 | 0.84 |  |  |  |
| full stomach | 2.372 | 0.973-5.78 | 0.057 |  |  |  |
| Histology | 0.551 | 0.259-1.173 | 0.122 |  |  |  |
| TNM stage |  |  |  |  |  |  |
| I | 1 |  | <0.001 |  |  | 0.458 |
| II | 2.202 | 0.637-7.608 | 0.212 |  |  | 0.999 |
| III | 5.542 | 2.188-14.037 | <0.001 |  |  | 0.292 |
| Chemotherapy | 2.037 | 1.115-3.723 | 0.021 |  |  | 0.513 |

HR, hazard ratio; CI, confidence interval; ALI, advanced lung cancer inflammation index; LPR, lymph node positive rate on biopsy; TNM, tumor-node-metastasis. ALI was grouped according to cutoff value (41.39) obtained from ROC curve. The reference of gender, age, ALI, tumor location, histology and TNM stage was male, age<65 years, low ALI, proximal stomach, undifferentiated and TNM stage I, respectively. LPR was analyzed as a continuous variable in univariate or multivariate analysis.

**Supplementary Table 5. Univariate and multivariate analyses for disease-free survival of male GC patients (N=469).**

| Parameters | Univariate analysis | | | Multivariate analysis | | |
| --- | --- | --- | --- | --- | --- | --- |
|  | HR | 95%CI | P value | HR | 95%CI | P value |
| Age | 1.884 | 1.411-2.514 | <0.001 | 1.913 | 1.419-2.578 | <0.001 |
| ALI | 0.553 | 0.416-0.736 | <0.001 | 0.664 | 0.494-0.892 | 0.007 |
| LPR | 8.987 | 5.829-13.856 | <0.001 | 4.604 | 2.764-7.667 | <0.001 |
| Tumor size | 1.183 | 1.124-1.246 | <0.001 |  |  | 0.111 |
| Tumor location |  |  |  |  |  |  |
| proximal stomach | 1 |  | 0.003 |  |  | 0.675 |
| distal stomach | 0.663 | 0.481-0.913 | 0.012 |  |  | 0.389 |
| full stomach | 1.22 | 0.808-1.841 | 0.345 |  |  | 0.568 |
| Histology | 0.854 | 0.632-1.156 | 0.307 |  |  |  |
| TNM stage |  |  |  |  |  |  |
| I | 1 |  | <0.001 | 1 |  | <0.001 |
| II | 1.882 | 0.881-4.022 | 0.103 | 2.614 | 1.132-6.036 | 0.024 |
| III | 6.699 | 3.942-11.386 | <0.001 | 8.403 | 4.171-16.936 | <0.001 |
| Chemotherapy | 2.497 | 1.76-3.543 | <0.001 | 0.476 | 0.302-0.75 | 0.001 |

HR, hazard ratio; CI, confidence interval; ALI, advanced lung cancer inflammation index; LPR, lymph node positive rate on biopsy; TNM, tumor-node-metastasis. ALI was grouped according to cutoff value (39.77) obtained from ROC curve. The reference of gender, age, ALI, tumor location, histology and TNM stage was male, age<65 years, low ALI, proximal stomach, undifferentiated and TNM stage I, respectively. LPR was analyzed as a continuous variable in univariate or multivariate analysis.

**Supplementary Table 6. Univariate and multivariate analyses for overall survival of GC patients after PSM (N=460).**

| Parameters | Univariate analysis | | | Multivariate analysis | | |
| --- | --- | --- | --- | --- | --- | --- |
|  | HR | 95%CI | P value | HR | 95%CI | P value |
| Gender | 1.005 | 0.711-1.42 | 0.978 |  |  |  |
| Age | 1.524 | 1.126-2.063 | 0.006 | 1.733 | 1.267-2.37 | 0.001 |
| ALI | 0.403 | 0.275-0.589 | <0.001 | 0.534 | 0.362-0.785 | 0.001 |
| LPR | 9.697 | 6.163-15.257 | <0.001 | 5.016 | 3.005-8.372 | <0.001 |
| Tumor size | 1.144 | 1.099-1.19 | <0.001 | 1.087 | 1.03-1.146 | 0.002 |
| Tumor location |  |  |  |  |  |  |
| proximal stomach | 1 |  | 0.005 |  |  |  |
| distal stomach | 0.76 | 0.535-1.08 | 0.126 |  |  |  |
| full stomach | 1.44 | 0.939-2.209 | 0.095 |  |  |  |
| Histology | 0.808 | 0.578-1.128 | 0.21 |  |  |  |
| TNM stage |  |  |  |  |  |  |
| I | 1 |  | <0.001 | 1 |  | 0.002 |
| II | 2.334 | 1.008-5.402 | 0.048 | 1.92 | 0.806-4.576 | 0.141 |
| III | 6.719 | 3.539-12.756 | <0.001 | 3.085 | 1.51-6.3 | 0.002 |
| Chemotherapy | 2.806 | 1.871-4.21 | <0.001 |  |  | 0.205 |

HR, hazard ratio; CI, confidence interval; ALI, advanced lung cancer inflammation index; LPR, lymph node positive rate on biopsy; TNM, tumor-node-metastasis. ALI was grouped according to cutoff value (19.08) obtained from ROC curve. The reference of gender, age, ALI, tumor location, histology and TNM stage was male, age<65 years, low ALI, proximal stomach, undifferentiated and TNM stage I, respectively. LPR was analyzed as a continuous variable in univariate or multivariate analysis.

**Supplementary Table 7. Univariate and multivariate analyses for overall survival of male GC patients after PSM (N=346).**

| Parameters | Univariate analysis | | | Multivariate analysis | | |
| --- | --- | --- | --- | --- | --- | --- |
|  | HR | 95%CI | P value | HR | 95%CI | P value |
| Age | 1.663 | 1.176-2.352 | 0.004 | 1.713 | 1.2-2.445 | 0.003 |
| ALI | 0.337 | 0.216-0.525 | <0.001 | 0.476 | 0.303-0.748 | 0.001 |
| LPR | 8.09 | 4.773-13.714 | <0.001 | 3.853 | 2.132-6.963 | <0.001 |
| Tumor size | 1.181 | 1.108-1.258 | <0.001 |  |  | 0.67 |
| Tumor location |  |  |  |  |  |  |
| proximal stomach | 1 |  | 0.044 |  |  |  |
| distal stomach | 0.723 | 0.487-1.073 | 0.107 |  |  |  |
| full stomach | 1.252 | 0.771-2.031 | 0.363 |  |  |  |
| Histology | 0.888 | 0.615-1.283 | 0.528 |  |  |  |
| TNM stage |  |  |  |  |  |  |
| I | 1 |  | <0.001 | 1 |  | <0.001 |
| II | 2.154 | 0.755-6.142 | 0.151 | 2.095 | 0.704-6.241 | 0.184 |
| III | 8.178 | 3.806-17.573 | <0.001 | 5.033 | 2.139-11.844 | <0.001 |
| Chemotherapy | 3.687 | 2.211-6.15 | <0.001 |  |  | 0.055 |

HR, hazard ratio; CI, confidence interval; ALI, advanced lung cancer inflammation index; LPR, lymph node positive rate on biopsy; TNM, tumor-node-metastasis. ALI was grouped according to cutoff value (18.27) obtained from ROC curve. The reference of gender, age, ALI, tumor location, histology and TNM stage was male, age<65 years, low ALI, proximal stomach, undifferentiated and TNM stage I, respectively. LPR was analyzed as a continuous variable in univariate or multivariate analysis.

**Supplementary Table 8. Univariate and multivariate analyses for overall survival of female GC patients after PSM (N=114).**

| Parameters | Univariate analysis | | | Multivariate analysis | | |
| --- | --- | --- | --- | --- | --- | --- |
|  | HR | 95%CI | P value | HR | 95%CI | P value |
| Age | 1.161 | 0.613-2.198 | 0.647 |  |  |  |
| ALI | 0.453 | 0.223-0.921 | 0.029 | 0.378 | 0.183-0.781 | 0.009 |
| LPR | 16.759 | 6.672-42.095 | <0.001 | 16.134 | 6.123-42.511 | <0.001 |
| Tumor size | 1.11 | 1.045-1.179 | 0.001 | 1.094 | 1.018-1.175 | 0.015 |
| Tumor location |  |  |  |  |  |  |
| proximal stomach | 1 |  | 0.043 |  |  |  |
| distal stomach | 0.955 | 0.414-2.202 | 0.913 |  |  |  |
| full stomach | 2.422 | 0.91-6.446 | 0.076 |  |  |  |
| Histology |  |  |  |  |  |  |
| TNM stage |  |  |  |  |  |  |
| I | 1 |  | 0.056 |  |  | 0.786 |
| II | 2.351 | 0.562-9.841 | 0.242 |  |  | 0.521 |
| III | 3.89 | 1.196-12.656 | 0.024 |  |  | 0.83 |
| Chemotherapy | 1.529 | 0.77-3.035 | 0.225 |  |  |  |

HR, hazard ratio; CI, confidence interval; ALI, advanced lung cancer inflammation index; LPR, lymph node positive rate on biopsy; TNM, tumor-node-metastasis. ALI was grouped according to cutoff value (24.07) obtained from ROC curve. The reference of gender, age, ALI, tumor location, histology and TNM stage was male, age<65 years, low ALI, proximal stomach, undifferentiated and TNM stage I, respectively. LPR was analyzed as a continuous variable in univariate or multivariate analysis.

**Supplementary Table 9. Univariate and multivariate analyses for disease-free survival of GC patients after PSM (N=460).**

| Parameters | Univariate analysis | | | Multivariate analysis | | |
| --- | --- | --- | --- | --- | --- | --- |
|  | HR | 95%CI | P value | HR | 95%CI | P value |
| Gender | 1.083 | 0.775-1.514 | 0.64 |  |  |  |
| Age | 1.536 | 1.151-2.05 | 0.004 | 1.754 | 1.302-2.362 | <0.001 |
| ALI | 0.522 | 0.369-0.738 | <0.001 | 0.599 | 0.409-0.878 | 0.009 |
| LPR | 8.846 | 5.718-13.685 | <0.001 | 4.655 | 2.834-7.648 | <0.001 |
| Tumor size | 1.143 | 1.099-1.187 | <0.001 | 1.09 | 1.036-1.146 | 0.001 |
| Tumor location |  |  |  |  |  |  |
| proximal stomach | 1 |  | 0.005 |  |  |  |
| distal stomach | 0.747 | 0.536-1.041 | 0.085 |  |  |  |
| full stomach | 1.369 | 0.908-2.066 | 0.134 |  |  |  |
| Histology | 0.844 | 0.616-1.156 | 0.29 |  |  |  |
| TNM stage |  |  |  |  |  |  |
| I | 1 |  | <0.001 | 1 |  | 0.002 |
| II | 2.11 | 0.992-4.49 | 0.053 | 1.723 | 0.794-3.739 | 0.169 |
| III | 5.688 | 3.229-10.017 | <0.001 | 2.684 | 1.432-5.03 | 0.002 |
| Chemotherapy | 2.708 | 1.85-3.964 | <0.001 |  |  | 0.273 |

HR, hazard ratio; CI, confidence interval; ALI, advanced lung cancer inflammation index; LPR, lymph node positive rate on biopsy; TNM, tumor-node-metastasis. ALI was grouped according to cutoff value (22) obtained from ROC curve. The reference of gender, age, ALI, tumor location, histology and TNM stage was male, age<65 years, low ALI, proximal stomach, undifferentiated and TNM stage I, respectively. LPR was analyzed as a continuous variable in univariate or multivariate analysis.

**Supplementary Table 10. Univariate and multivariate analyses for disease-free survival of male GC patients after PSM (N=346).**

| Parameters | Univariate analysis | | | Multivariate analysis | | |
| --- | --- | --- | --- | --- | --- | --- |
|  | HR | 95%CI | P value | HR | 95%CI | P value |
| Age | 1.603 | 1.154-2.228 | 0.005 | 1.745 | 1.241-2.453 | 0.001 |
| ALI | 0.38 | 0.245-0.591 | <0.001 | 0.571 | 0.363-0.896 | 0.015 |
| LPR | 7.243 | 4.377-11.986 | <0.001 | 3.118 | 1.735-5.601 | <0.001 |
| Tumor size | 1.175 | 1.107-1.248 | <0.001 | 1.08 | 1.006-1.16 | 0.032 |
| Tumor location |  |  |  |  |  |  |
| proximal stomach | 1 |  | 0.081 |  |  |  |
| distal stomach | 0.743 | 0.513-1.077 | 0.117 |  |  |  |
| full stomach | 1.178 | 0.737-1.881 | 0.494 |  |  |  |
| Histology | 0.926 | 0.656-1.308 | 0.664 |  |  |  |
| TNM stage |  |  |  |  |  |  |
| I | 1 |  | <0.001 | 1 |  | <0.001 |
| II | 1.955 | 0.794-4.813 | 0.145 | 1.628 | 0.641-4.135 | 0.305 |
| III | 6.362 | 3.334-12.138 | <0.001 | 3.42 | 1.655-7.067 | 0.001 |
| Chemotherapy | 3.319 | 2.087-5.28 | <0.001 |  |  | 0.745 |

HR, hazard ratio; CI, confidence interval; ALI, advanced lung cancer inflammation index; LPR, lymph node positive rate on biopsy; TNM, tumor-node-metastasis. ALI was grouped according to cutoff value (18.27) obtained from ROC curve. The reference of gender, age, ALI, tumor location, histology and TNM stage was male, age<65 years, low ALI, proximal stomach, undifferentiated and TNM stage I, respectively. LPR was analyzed as a continuous variable in univariate or multivariate analysis.

**Supplementary Table 11. Univariate and multivariate analyses for disease-free survival of female GC patients after PSM (N=114).**

| Parameters | Univariate analysis | | | Multivariate analysis | | |
| --- | --- | --- | --- | --- | --- | --- |
|  | HR | 95%CI | P value | HR | 95%CI | P value |
| Age | 1.332 | 0.723-2.455 | 0.357 |  |  |  |
| ALI | 0.486 | 0.24-0.982 | 0.044 | 0.409 | 0.2-0.838 | 0.015 |
| LPR | 17.459 | 6.935-43.956 | <0.001 | 16.801 | 6.343-44.501 | <0.001 |
| Tumor size | 1.111 | 1.048-1.178 | <0.001 | 1.097 | 1.024-1.175 | 0.009 |
| Tumor location |  |  |  |  |  |  |
| proximal stomach | 1 |  | 0.018 |  |  |  |
| distal stomach | 0.931 | 0.376-1.837 | 0.647 |  |  |  |
| full stomach | 2.322 | 0.922-5.843 | 0.074 |  |  |  |
| Histology | 0.457 | 0.18-1.16 | 0.099 |  |  |  |
| TNM stage |  |  |  |  |  |  |
| I | 1 |  | 0.037 |  |  | 0.8 |
| II | 2.367 | 0.566-9.908 | 0.238 |  |  | 0.64 |
| III | 4.184 | 1.289-13.58 | 0.017 |  |  | 0.95 |
| Chemotherapy | 1.649 | 0.835-3.258 | 0.15 |  |  |  |

HR, hazard ratio; CI, confidence interval; ALI, advanced lung cancer inflammation index; LPR, lymph node positive rate on biopsy; TNM, tumor-node-metastasis. ALI was grouped according to cutoff value (24.07) obtained from ROC curve. The reference of gender, age, ALI, tumor location, histology and TNM stage was male, age<65 years, low ALI, proximal stomach, undifferentiated and TNM stage I, respectively. LPR was analyzed as a continuous variable in univariate or multivariate analysis.

**Supplementary Figure S1.** ROC analysis for the prediction of OS. AUC indicates the diagnostic power of ALI (A) in overall series, (B) in female subgroup and (C) in male subgroup. AUC indicates the diagnostic power of BMI (D) in overall series, (E) in female subgroup and (F) in male subgroup. AUC indicates the diagnostic power of ALB (G) in overall series, (H) in female subgroup and (I) in male subgroup. AUC indicates the diagnostic power of NLR (J) in overall series, (K) in female subgroup and (L) in male subgroup. Respective cutoff values obtained at the maximum Youden index was used to divide them into two groups. ROC, receiver operating characteristic curve; OS, overall survival; AUC, area under the ROC curve; ALI, advanced lung cancer inflammation index; BMI, body mass index; ALB, albumin; NLR, neutrophil-lymphocyte ratio.


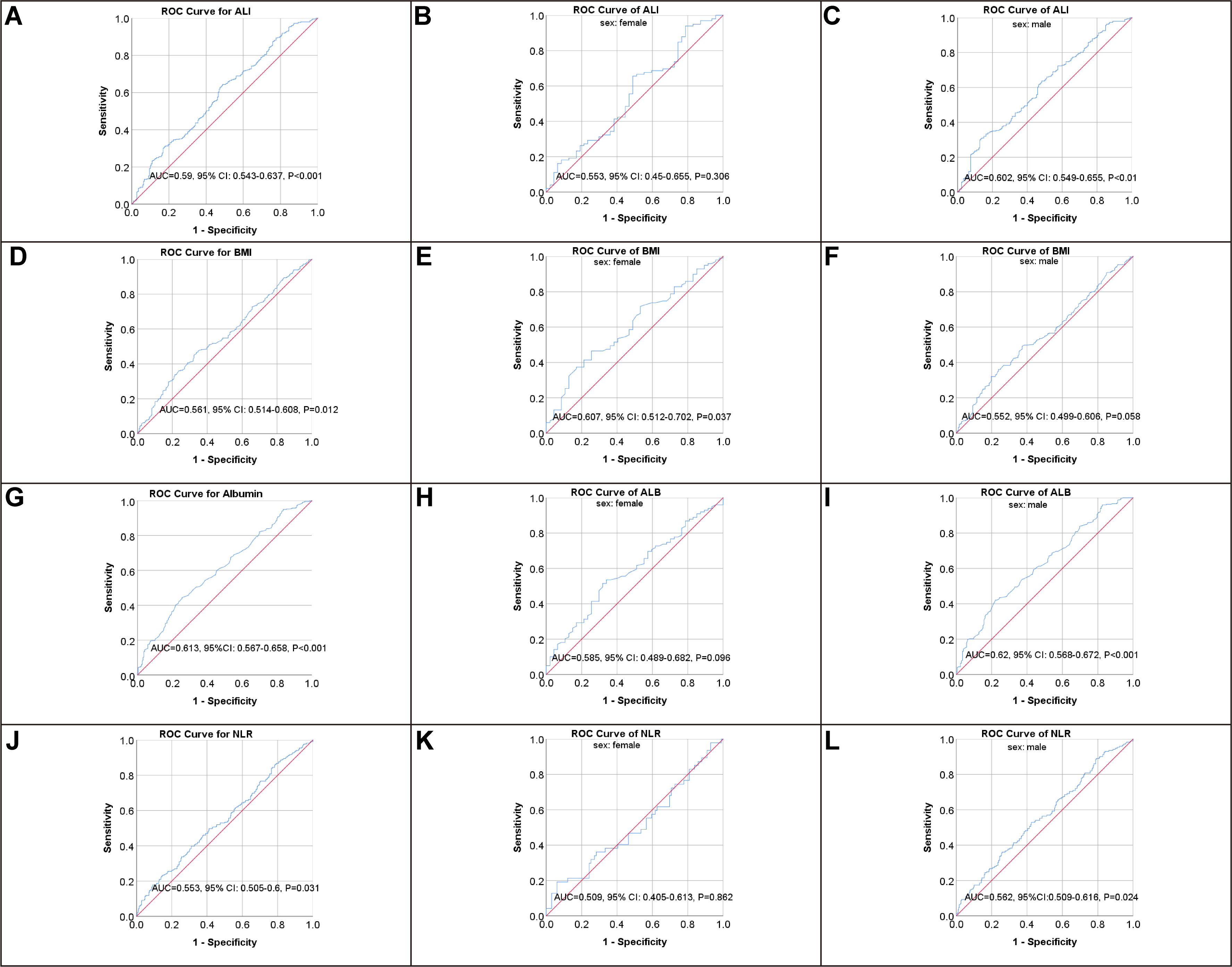


**Supplementary Figure S2.** ROC analysis for the prediction of DFS. AUC indicates the diagnostic power of ALI (A) in overall series, (B) in female subgroup and (C) in male subgroup. AUC indicates the diagnostic power of BMI (D) in overall series, (E) in female subgroup and (F) in male subgroup. AUC indicates the diagnostic power of ALB (G) in overall series, (H) in female subgroup and (I) in male subgroup. AUC indicates the diagnostic power of NLR (J) in overall series, (K) in female subgroup and (L) in male subgroup. Respective cutoff values obtained at the maximum Youden index was used to divide them into two groups. DFS, disease-free survival.


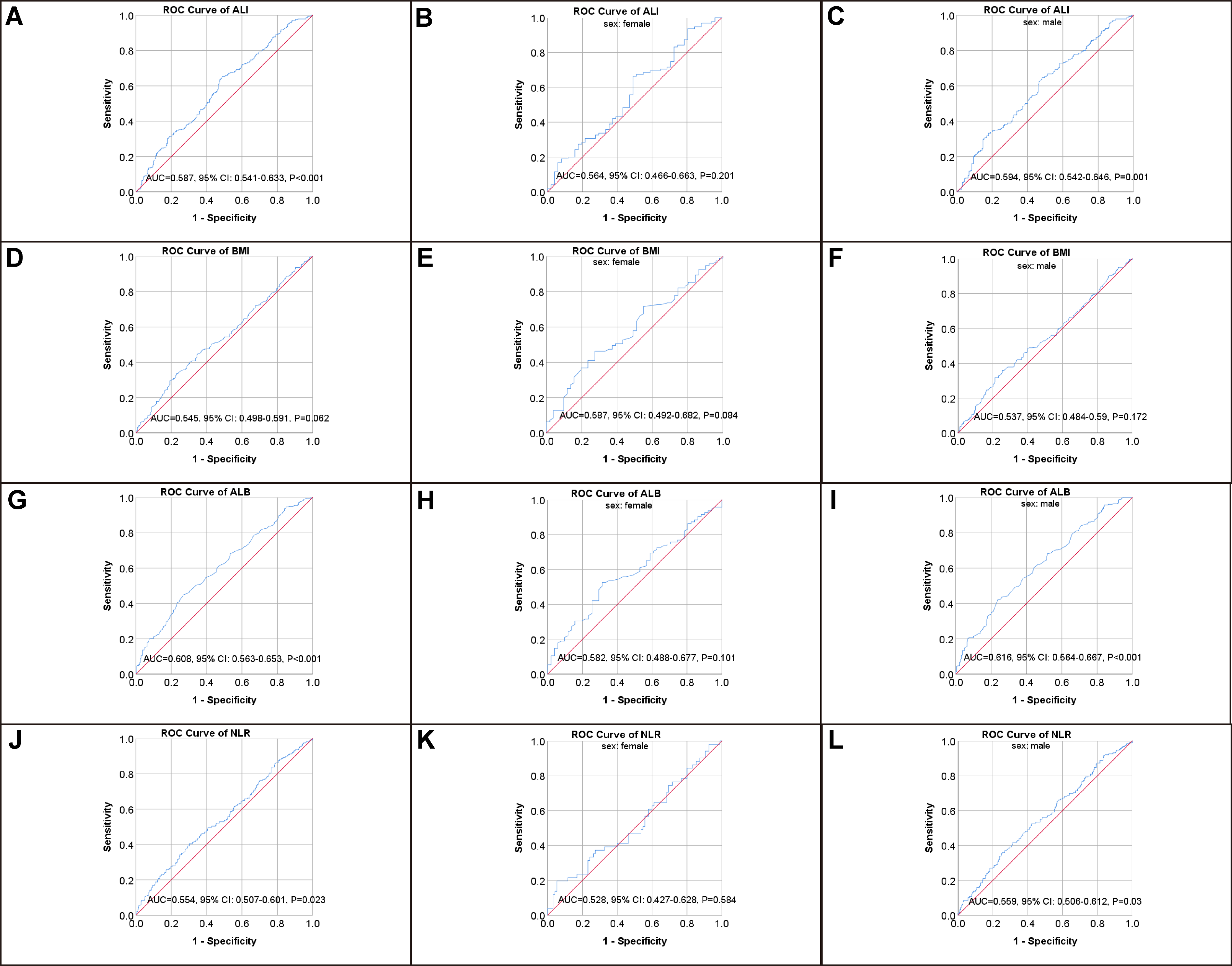


**Supplementary Figure S3.** Kaplan-Meier survival analysis of BMI, ALB and NLR for the prediction of OS. Kaplan-Meier survival curves of BMI (A) in all patients, (B) in female patients and (C) in male patients. Kaplan-Meier survival curves of ALB (D) in all patients, (E) in female patients and (F) in male patients. Kaplan-Meier survival curves of NLR (G) in all patients, (H) in female patients and (I) in male patients. The respective cutoff values were obtained from the ROC curves of the subgroups. Both cutoff values and statistical significance are shown in figure. (J-L) Kaplan-Meier survival curves of BMI in groups stratified by TNM stage (P=0.205, 0.344 and 0.047, respectively).


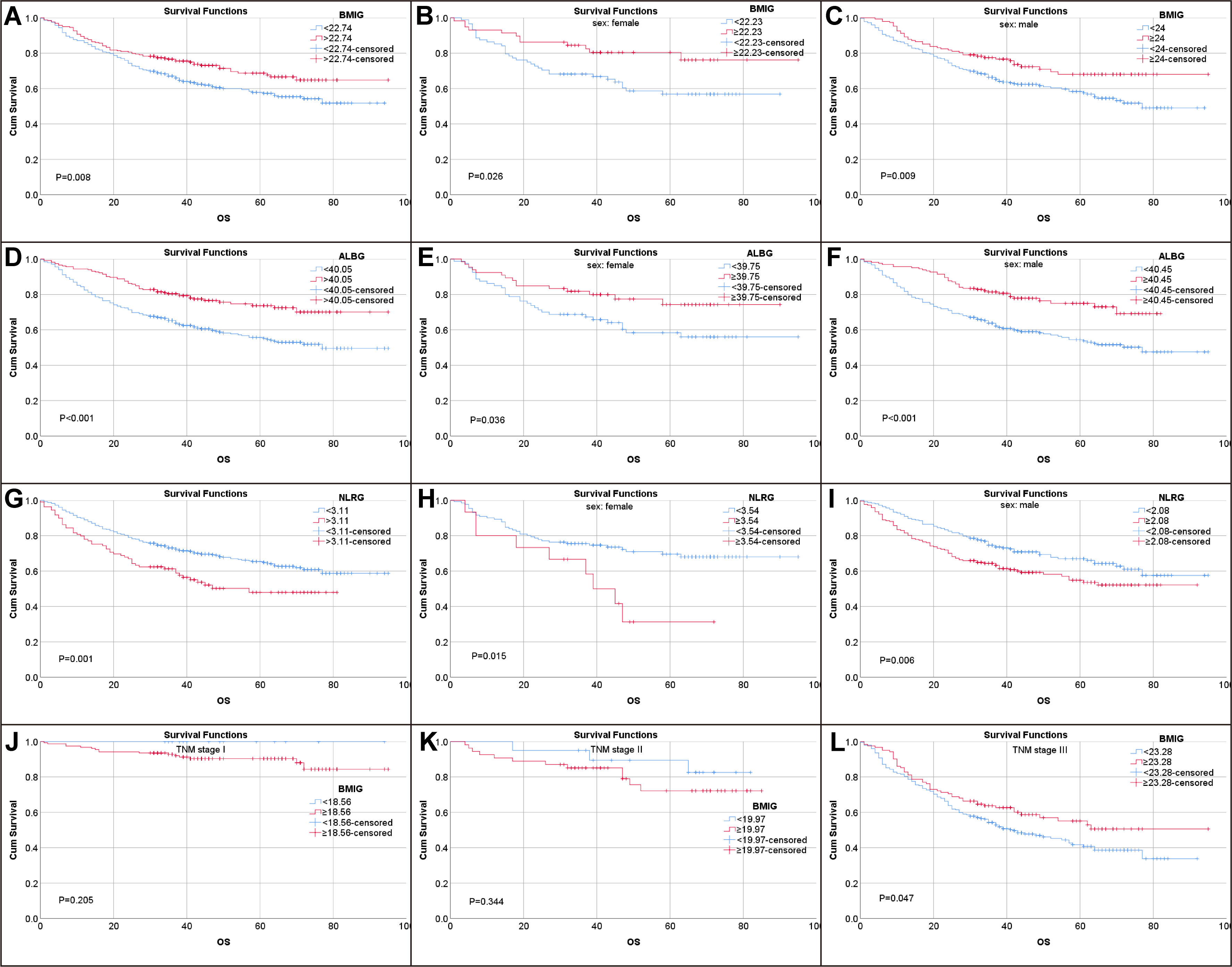


**Supplementary Figure S4.** Kaplan-Meier survival analysis of BMI, ALB and NLR for the prediction of DFS. Kaplan-Meier survival curves of BMI (A) in all patients, (B) in female patients and (C) in male patients. Kaplan-Meier survival curves of ALB (D) in all patients, (E) in female patients and (F) in male patients. Kaplan-Meier survival curves of NLR (G) in all patients, (H) in female patients and (I) in male patients. The respective cutoff values were obtained from the ROC curves of the subgroups and shown in figure. (J-L) Kaplan-Meier survival curves of BMI in groups stratified by TNM stage (P=0.229, 0.211 and 0.047, respectively).


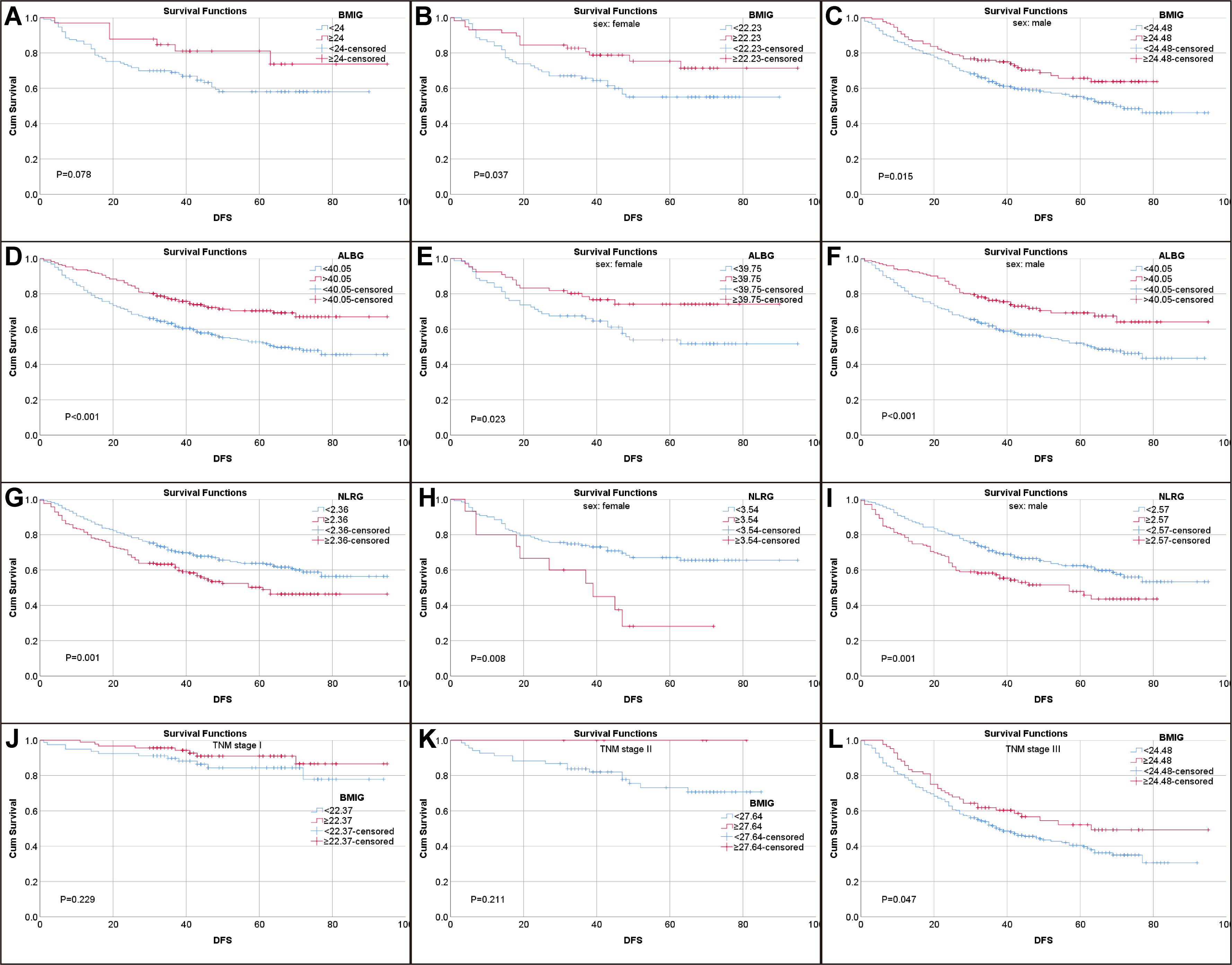


**Supplementary Figure S5.** ROC analysis for the prediction of OS and DFS in dataset after PSM. (A) ROC curve of ALI for the prediction of OS of all patients in this dataset; (B) ROC curve of ALI for the prediction of DFS of all patients in this dataset; (C) ROC curve of ALI for the prediction of OS of male patients; (D) ROC curve of ALI for the prediction of DFS of male patients; (E) ROC curve of ALI for the prediction of OS of female patients; (F) ROC curve of ALI for the prediction of DFS of female patients.


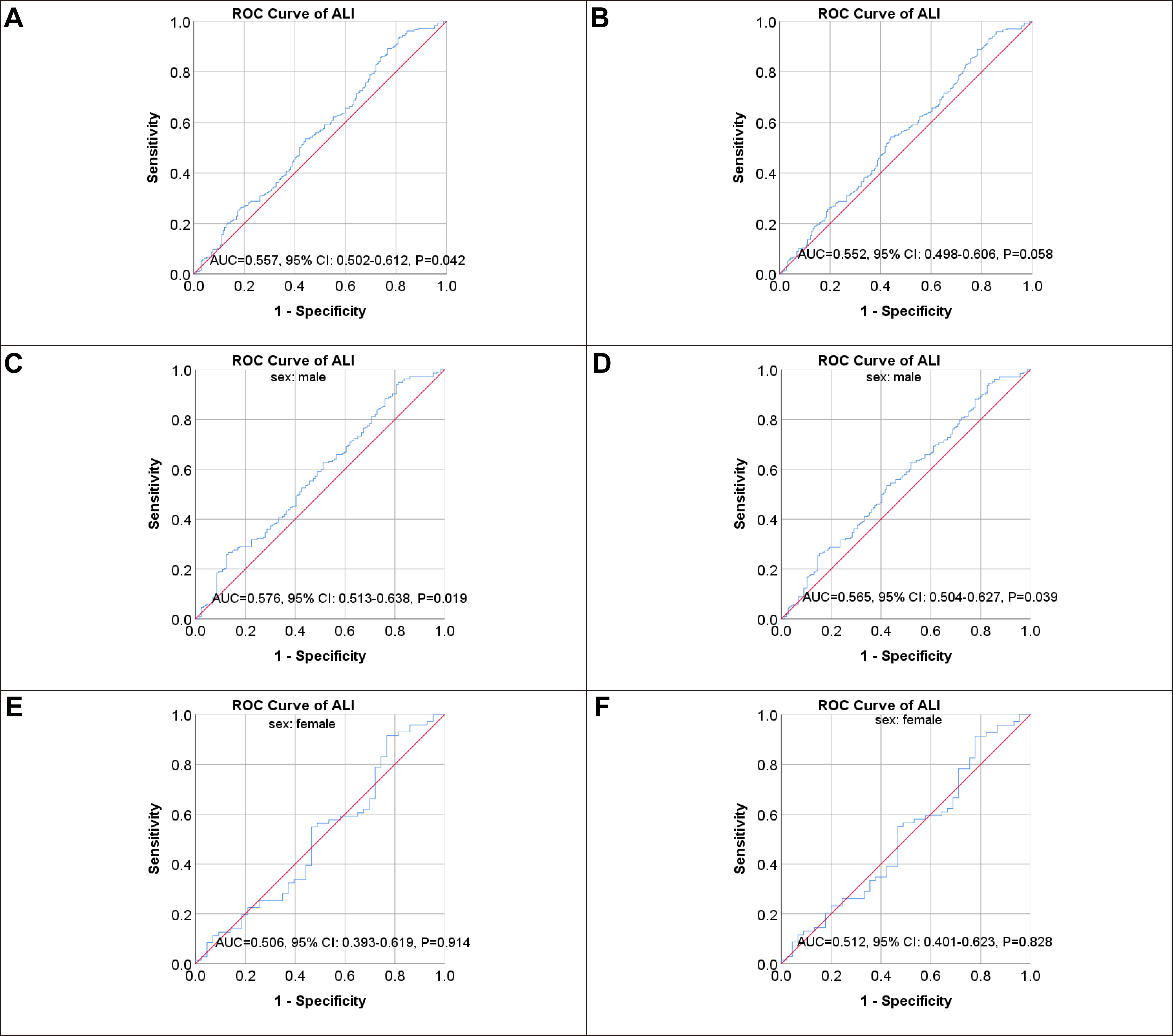

Supplement: Supplementary file 1 — Additional file 1: Table S1. Cutoff values for parameters in datasets before and after PSM. Table S2. Univariate and multivariate analyses for overall survival of female GC patients (N=146). Table S3. Univariate and multivariate analyses for overall survival of male GC patients (N=469). Table S4. Univariate and multivariate analyses for disease-free survival of female GC patients (N=146). Table S5. Univariate and multivariate analyses for disease-free survival of male GC patients (N=469). Table S6. Univariate and multivariate analyses for overall survival of GC patients after PSM (N=460). Table S7. Univariate and multivariate analyses for overall survival of male GC patients after PSM (N=346). Table S8. Univariate and multivariate analyses for overall survival of female GC patients after PSM (N=114). Table S9. Univariate and multivariate analyses for disease-free survival of GC patients after PSM (N=460). Table S10. Univariate and multivariate analyses for disease-free survival of male GC patients after PSM (N=346). Table S11. Univariate and multivariate analyses for disease-free survival of female GC patients after PSM (N=114). Figure S1. ROC analysis for the prediction of OS. AUC indicates the diagnostic power of ALI (A) in overall series, (B) in female subgroup and (C) in male subgroup. AUC indicates the diagnostic power of BMI (D) in overall series, (E) in female subgroup and (F) in male subgroup. AUC indicates the diagnostic power of ALB (G) in overall series, (H) in female subgroup and (I) in male subgroup. AUC indicates the diagnostic power of NLR (J) in overall series, (K) in female subgroup and (L) in male subgroup. Respective cutoff values obtained at the maximum Youden index was used to divide them into two groups. ROC, receiver operating characteristic curve; OS, overall survival; AUC, area under the ROC curve; ALI, advanced lung cancer inflammation index; BMI, body mass index; ALB, albumin; NLR, neutrophil-lymphocyte ratio. Figure S2. ROC analysis for t [file 12885_2022_9774_MOESM1_ESM.doc]
